# Measuring What Matters: Particle–Chemical Domains and Analytical Gaps in Biomonitoring of Micro- and Nanoplastics

**DOI:** 10.1021/acs.est.6c04283

**Published:** 2026-05-05

**Authors:** Vittorio Albergamo, Miguel A. Modestino, Leonardo Trasande

**Affiliations:** † Department of Pediatrics, Division of Environmental Pediatrics, 12296 NYU Grossman School of Medicine, New York, New York 10016, United States; ‡ Department of Chemical and Biomolecular Engineering, NYU Tandon School of Engineering, Brooklyn, New York 11201, United States; § NYU Department of Population Health, New York, New York 10016, United States; ∥ NYU Wagner School of Public Services, New York, New York 10003, United States

**Keywords:** plastic pollution, environmental health, public health, microplastics, nanoplastics

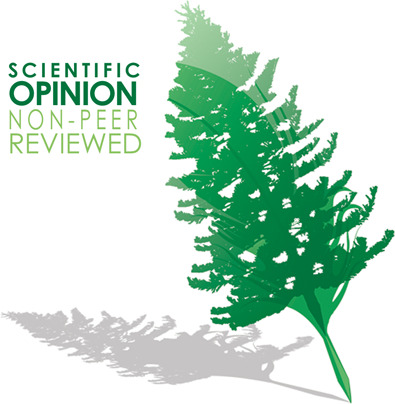

## Graphical abstract

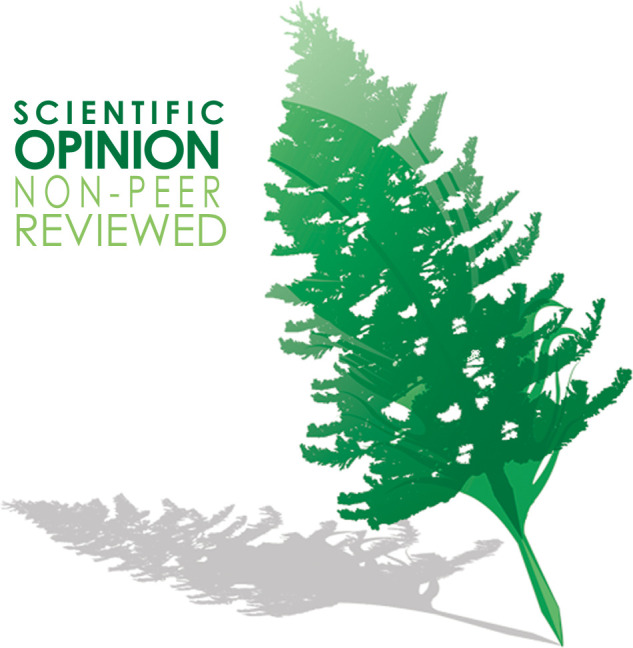

The trajectory is clear: plastic pollution is an intersecting environmental and public health challenge that warrants urgent attention.^1,2^ Within this broader concern, increasing attention is being paid to microplastics and nanoplastics (MNPs): small solid plastic particles that contaminate air, water, food, and indoor environments and are now detected in multiple human tissues and excreta in a small but growing number of studies.^3^ MPs are defined as polymer particles <5 mm, either primary (intentionally manufactured) or secondary, generated by environmental degradation of larger plastic debris. Continued breakdown of these fragments, together with certain industrial and combustion processes, yields nanoplastics (NPs), generally considered to have at least one dimension <1 μm, although definitions vary. Given the vast and still growing accumulation of plastic waste in the environment, the burden of MNPs on ecosystems—and with it human exposure—is poised to rise substantially.^4^

## The Two-Domain Issue

Within the human exposome framework, MNPs represent a uniquely complex exposure class: unlike most environmental contaminants, they simultaneously implicate two distinct domains, a physical particle domain and a chemical domain, both of which must be considered alongside internal biological responses to fully capture their contribution to cumulative human health risk. The physical particle domain comprises MNPs as solid materials whose size, shape, microstructure, and surface properties govern uptake, translocation, and toxicity.^5^ The chemical domain comprises the complex mixture of (co)­monomers and how they are bonded, processing aids, intentionally added additives (IAS), nonintentionally added substances (NIAS), and sorbed environmental contaminants. Many of these substances are not covalently bound and can leach or desorb, acting as independent chemical toxicants that are carried by MNPs. Recent mapping of the PlastChem inventory identified 16,325 distinct plastic chemicals, including 5,776 additives, 3,498 processing aids, 1,975 starting substances, and 1,788 NIAS; more than 4,200 of these—roughly one in four—meet hazard-based criteria as chemicals of concern (*e.g.*, persistence, bioaccumulation, or toxicity).^6^

In both scientific debate and public communication, the term “microplastics toxicity” often blurs these two domains, making it difficult to know whether observed effects arise from particle properties, from associated chemicals, or from their combination.

Adverse health effects from the chemical domain are already well established: key IAS classes such as phthalates, bisphenols, PFAS, and flame retardants are linked to endocrine and neurodevelopmental toxicity, metabolic disease, reproductive dysfunction, and increased risk of some cancers,^7^ with quantifiable disease and economic burdens.^8^ By contrast, particle-driven effects in human populations remain poorly understood, and largely associative, highlighting a critical gap that targeted research efforts must address.

## Analytical Bottlenecks in Biomonitoring of Micro- and Nanoplastics

A key step toward understanding how MNPs affect human health *in vivo* is to overcome current analytical limitations. Looking at the regulatory foundations for analysis of organic chemicals, it is evident that many of the same principles for method development and validation, quality control, and interlaboratory comparability could and should be adapted to guide MNP biomonitoring.

From sample collection through data acquisition, MNP analysis in human biospecimens lacks standardized procedures. Sampling protocols remain heterogeneous and often face challenging constraints to limit ambient contamination, including in clinic or hospital settings. MNP extraction from biological samples typically requires matrix digestion before particle separation, with procedures using a wide array of reagents and conditions, including strong acids, bases, and oxidative agents such as hydrogen peroxide or Fenton’s reagent. These approaches can alter particle’s surface chemistry, recovery, and induce fragmentation. Furthermore, MNPs may agglomerate in aqueous digestion media, which can bias size distribution measurements and complicate identification by spectroscopic methods, especially where instrumental sensitivity differs across polymer types. Separation approaches typically rely on sedimentation, filtration, or combinations of both. Smaller NPs cannot sediment without ultracentrifugation because their motion is dominated by viscous drag, so researchers must either use polymeric centrifuge tubes, which risk contamination and sorptive losses, or accept incomplete recovery of nanosized particles. Filtration-based workflows face a similar trade-off: very small pore sizes increase the risk of filter clogging and slow filtration, whereas larger pore sizes systematically lose NPs. Beyond these analytical workflow steps, the lack of suitable reference materials further limits method development and validation. Environmentally relevant standard mixtures of MNPs that reflect their size, shape, polymer types, and chemical status (*e.g.*, IAS/NIAS content, degree of degradation) are not readily available, and internal standards to assess procedural losses do not yet exist for most plastic polymers or key additives. As a result, thorough bioanalytical method development and validation remain difficult. These limitations directly constrain epidemiological research, precluding harmonized exposure metrics, pooled analyses, and causal inference across studies.

The analytical toolbox for organic chemicals associated with plastics is comparatively mature. For IAS and NIAS, tandem mass spectrometry-based methods leveraging gas chromatography (GC-MS/MS) and liquid chromatography (LC-MS/MS) represent a well-established gold standard for quantification, while high-resolution mass spectrometry-based screenings (GC-HRMS and LC-HRMS) enable nontarget identification and semiquantification. Yet, due to the sheer number of substances involved, comprehensive characterization of the MNP chemical domain remains a substantial challenge. Particle detection and characterization in biospecimens has no equivalent gold standard, and fundamental limitations persist across the entire analytical workflow. No single analytical technique can simultaneously characterize particle number concentration, morphology, polymer mass and composition, and physicochemical properties alongside the chemicals MNPs carry. Investigators often must choose which properties to prioritize across the two domains instead of relying on a single or a set of comprehensive assays. If the key question is body burden for dose–response (*e.g.*, epidemiology), mass-based metrics are critical. If the question is particle-driven mechanisms (*e.g.*, translocation, local inflammation), size and shape distributions are more informative. If the question concerns hazard characterization of the chemical domain, nontarget screening with hyphenated HRMS should accompany targeted quantification. Fit-for-purpose measurement requires explicit justification of the selected metrics relative to the research question, rigorous contamination controls, transparent reporting of polymer particle size ranges and types, and chemical identification confidence levels,^9^ and (semiquantitative) concentrations to enable interstudy comparability.

A small set of complementary techniques has become the backbone of MNP biomonitoring: Raman microspectroscopy (μ-Raman; based on inelastic scattering of narrowband light probing molecular vibrations), Fourier-transform infrared spectroscopy (μ-FTIR; based on absorbance of broadband infrared light by molecular bonds), laser direct infrared (LDIR; combines laser-based particle detection with infrared spectroscopy to determine particle size and chemical identity), and pyrolysis–gas chromatography–mass spectrometry (Py-GC/MS; the dominant tool for per-polymer mass quantification, leveraging thermal degradation of polymers to yield characteristic chemical markers identifiable by mass spectrometry). Figure 1 summarizes how these analytical platforms map onto key particle, interdomain, and chemical domains, and highlights their respective particle-size detection limits where applicable. Although platforms capable of probing NPs exist (*e.g.*, O-PTIR, hyperspectral dark-field microscopy, SERS-enhanced Raman), they remain largely underrepresented in current human biomonitoring and therefore are not discussed here. Each technique captures only a subset of the particle and chemical domains, reinforcing the need for complementary measurement strategies, *e.g.*, combining nondestructive vibrational spectroscopy techniques with mass quantification by Py-GC/MS, while repurposing the digested matrix for chemical analysis. Beyond identification and quantification, complementary polymer characterization techniques—including methods sensitive to properties such as chain length, topology, and crystallinity—can reveal physicochemical characteristics linked to hazard potential, providing a foundation for both risk prioritization and efforts toward safer material design.

**Figure 1:**
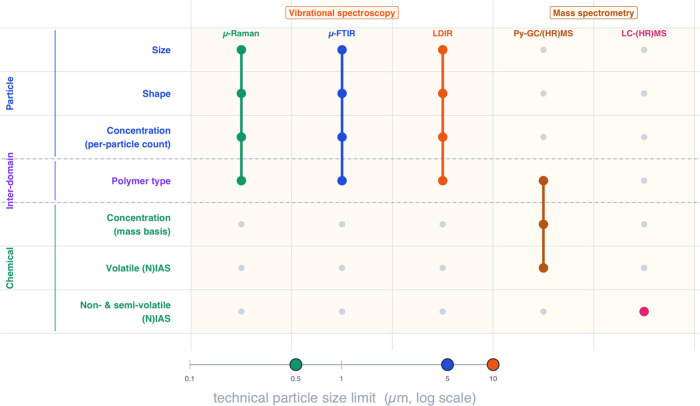
Analytical capabilities of complementary techniques for micro- and nanoplastic characterization across the particle and chemical domains. Techniques span vibrational spectroscopy (μ-Raman, μ-FTIR, and LDIR) and mass spectrometry platforms [Py-GC/(HR)­MS and LC-(HR)­MS]. The bottom scale shows approximate technical particle-size detection limits (logarithmic micrometer axis), with marker colors matching the techniques above.

Put bluntly: without comprehensive and rigorous measurement of both particles and chemicals, our evidence on plastic hazards will remain incomplete, may understate the true risks, and will slow down the development of novel, less-hazardous materials. Moving forward, the field must prioritize three coordinated actions: adopting fit-for-purpose measurement frameworks matched to the research question at hand; establishing minimum reporting standards now, ahead of fully harmonized methods, to enable the cross-study synthesis that robust epidemiological studies demand; and investing in environmentally relevant reference materials and nanoplastic-capable analytical platforms to close the measurement gap at the lower end of the particle size spectrum. Our analytical capabilities must grow to meet the scale of this environmental and public health challenge.
